# C‐Reactive Protein–Albumin–Lymphocyte Index as a Predictor of All‐Cause and Cardiovascular Mortality in Individuals With Diabetes or Prediabetes: A National Population‐Based Cohort Study

**DOI:** 10.1155/ije/2774096

**Published:** 2026-02-09

**Authors:** Qian Liu, Renyang Liu, Jing Yang, Jie Li

**Affiliations:** ^1^ Department of Neurology, Xiangyang Central Hospital, Affiliated Hospital of Hubei University of Arts and Science, Xiangyang, Hubei, China, xfszxyy.cn; ^2^ Department of Orthopaedics, Xiangyang Central Hospital, Affiliated Hospital of Hubei University of Arts and Science, Xiangyang, Hubei, China, xfszxyy.cn

**Keywords:** C-reactive protein–albumin–lymphocyte, diabetes, mortality, national health and nutrition examination, prediabetes

## Abstract

**Background:**

The C‐reactive protein–albumin–lymphocyte (CALLY) index is a novel composite marker that reflects systemic inflammation, nutrition, and immune status. However, its association with mortality among individuals with diabetes or prediabetes remains uncertain. This study aimed to assess the relationship between the CALLY index and both all‐cause (ACM) and cardiovascular mortality (CVM) in U.S. adults with diabetes or prediabetes.

**Methods:**

A total of 8463 adults with diabetes or prediabetes from the U.S. NHANES (2003–2010 and 2015–2018) were included in the analysis. Cox proportional hazards models were used to evaluate potential linear associations between the CALLY index and ACM and CVM. Kaplan–Meier survival curves and log‐rank tests were used to compare cumulative survival across groups. Restricted cubic spline (RCS) models were applied to examine potential nonlinear associations.

**Results:**

During an average follow‐up of 7.83 years, 1391 participants died, 470 from cardiovascular causes. After adjusting for all confounders, the natural log‐transformed (ln) CALLY index was inversely associated with ACM (HR = 0.83, 95% CI: 0.79–0.88) and CVM (HR = 0.82, 95% CI: 0.74–0.92). Compared to participants in the lowest quartile of the ln CALLY index, those in the highest quartile had significantly lower risks of ACM (HR = 0.67, 95% CI: 0.55–0.81) and CVM (HR = 0.66, 95% CI: 0.45–0.98). Kaplan–Meier survival analysis showed significantly higher survival probabilities among individuals in higher ln CALLY index quartiles (*p* < 0.001). RCS models further indicated a nonlinear relationship between the ln CALLY index and both ACM and CVM (*p* < 0.0001 for nonlinearity).

**Conclusions:**

A higher CALLY index is independently associated with lower risks of ACM and CVM among individuals with diabetes or prediabetes. These findings suggest that the CALLY index may serve as a valuable marker for monitoring mortality risk in these populations.

## 1. Introduction

Diabetes is a leading cause of death and disability worldwide, posing a serious public health challenge. Current projections indicate that the worldwide prevalence of diabetes will rise to 1.31 billion individuals by 2050 [1]. Extensive research has demonstrated that chronic hyperglycemia and insulin resistance—key pathophysiological features of diabetes—significantly elevate the risk of both microvascular complications (e.g., retinopathy, nephropathy) and macrovascular complications (e.g., stroke, coronary heart disease) [2–4]. Furthermore, individuals with prediabetes are also at a long‐term risk of progressing to diabetes and developing related complications [5]. Consequently, all‐cause mortality (ACM) and cardiovascular mortality (CVM) rates are significantly elevated among individuals with diabetes and prediabetes [6]. Early identification of high‐risk factors is therefore essential for timely intervention and improved prognosis in these populations.

It is well established that diabetes and its complications are associated with multiple interrelated factors, including inflammation, nutrition, and immune status [7, 8]. Studies have also explored the complex relationships between these factors and adverse outcomes such as vascular complications and mortality in individuals with diabetes. For example, hyperglycemia and insulin resistance can induce endothelial dysfunction and inflammatory responses, contributing to atherosclerotic lesions in the cardiovascular and cerebrovascular systems [9, 10]. Elevated levels of inflammatory markers, such as C‐reactive protein (CRP), predict vascular complications, and mortality in diabetic patients [11–15]. In addition, malnutrition is common among elderly diabetic individuals [16] and is strongly associated with increased mortality across diverse pathologies [17]. Serum albumin reflects both nutritional status and inflammatory burden [18], and has been reported to be closely associated with cardiovascular disease (CVD) and mortality [19]. Furthermore, hyperglycemia can lead to immune dysfunction by activating proinflammatory responses [20]. While B lymphocytes contribute to the chronic inflammatory state associated with diabetes by secreting proinflammatory cytokines and enhancing the function of proinflammatory T cells [21], lymphocyte count has been shown to correlate with CVD risk [22]. However, the limitations of single biomarkers in assessing disease risk have led to their not being widely adopted, and a multidimensional composite index is needed to integrate inflammation, nutrition, and immune status.

Lida *et al.* proposed the C‐reactive protein–albumin–lymphocyte (CALLY) index as a comprehensive marker reflecting systemic inflammation, nutrition, and immune status [23]. This index has been widely used to evaluate treatment prognosis in patients with malignancies [24–26]. In addition, recent studies have reported that the CALLY index is closely associated with both ACM and CVM in various populations, including older adults in the United States, patients with chronic obstructive pulmonary disease, individuals with CVD, and elderly cohorts in China [27–30]. The interplay among inflammation, nutrition, and immune status plays a critical role in the pathophysiology of diabetes. However, the association between the CALLY index and mortality risk in individuals with diabetes or prediabetes remains unexplored.

This study aimed to conduct a nationally representative cohort analysis to investigate the association between the CALLY index and the risks of ACM and CVM among individuals with diabetes or prediabetes.

## 2. Method

### 2.1. Data Source

NHANES applies a stratified, multistage probability sampling strategy to obtain detailed and nationally representative information on the nutritional and health conditions of the U.S. population [31]. NHANES was approved by the NCHS Research Ethics Review Board, and all participants provided written informed consent. Therefore, no additional ethical approval was required for this study. Further details are available at https://www.cdc.gov/nchs/nhanes/index.htm.

### 2.2. Study Population

**Figure 1:**
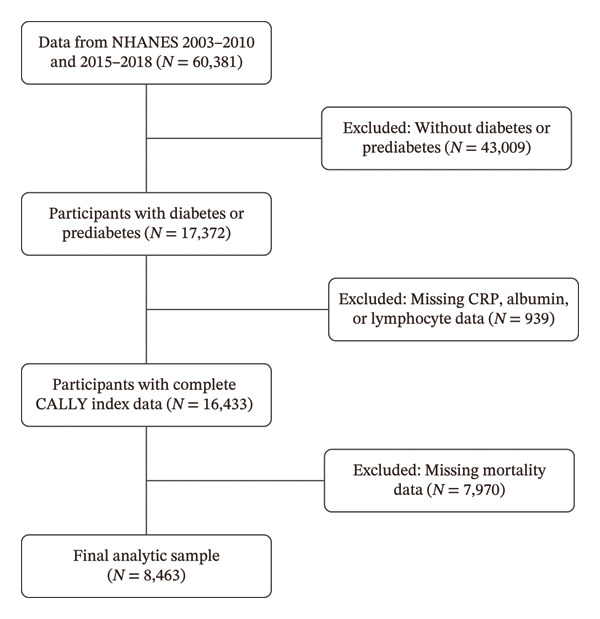
Flowchart of the participants’ selection from NHANES 2003–2010 and 2015–2018. Abbreviations: CALLY, C‐reactive protein–albumin–lymphocyte index; CRP, C‐reactive protein.

This study analyzed data from 60,381 participants across 6 NHANES cycles, covering the period from 2003 to 2010 and 2015 to 2018. Participants not meeting the 2021 American Diabetes Association criteria for diabetes or prediabetes were excluded (*n* = 43,009) [32]. Participants missing any component of the CALLY index (serum CRP, serum albumin, and lymphocyte counts from whole blood) (*n* = 939) or mortality data (*n* = 7970) were excluded, leaving 8,463participants in the final analysis (Figure 1).

### 2.3. Definitions of Outcome Variables

Mortality data were obtained from death certificates linked through the National Death Index (https://www.cdc.gov/nchs/data-linkage/mortality-recordspublic.htm), with follow‐up from the date of the NHANES examination until death or December 31, 2019, whichever came first. All‐cause and CVD‐related mortality were assessed in this study, with causes of death classified according to the International Classification of Diseases, 10th Revision (ICD‐10). CVD mortality included deaths due to rheumatic heart disease (I00–I09), hypertensive heart disease (I11–I13), ischemic heart disease (I20–I25), other heart diseases (I26–I51), and cerebrovascular diseases (I60–I69) [International Classification of Diseases. Available from: https://iris.who.int/handle/10665/246208].

### 2.4. Definitions of Exposure Variables

Blood samples were collected from participants at mobile examination centers and subsequently analyzed at a central laboratory to determine CRP, albumin, and lymphocyte levels, following standardized procedures. Lymphocyte counts were derived from complete blood count (CBC) parameters measured using the Beckman Coulter automated counting and quantification system. Serum albumin was measured using the Roche Cobas 6000 analyzer with the bromocresol purple method [33]. Serum CRP concentrations were determined via latex‐enhanced nephelometry, with the Behring Nephelometer used for data from NHANES 2003 to 2010, and the Beckman Coulter Synchron analyzer used for data from NHANES 2015 to 2018 [34]. All assays (lymphocytes, CRP, and albumin) were calibrated according to standardized procedures and participated in CDC external quality assurance programs, with performance meeting predefined accuracy and precision criteria. Laboratory procedures are described at https://wwwn.cdc.gov/nchs/nhanes/continuousnhanes/labmethods.aspx.The. The CALLY index was subsequently calculated as (serum albumin [g/dL] × absolute lymphocyte count [cells/L])/(CRP [mg/dL] × 10^4^ [35]. Given the skewed distribution of the CALLY index, a natural logarithmic transformation was applied to normalize the data. Both the continuous form and a categorical form—divided into quartiles—were used in the statistical analysis.

### 2.5. Assessment of Covariates

Covariates were selected based on prior studies linking them to ACM and CVM. Demographic variables included age, sex, race/ethnicity, education level, marital status, and the poverty income ratio (PIR). Behavioral and physical examination factors included body mass index (BMI), alcohol consumption (≥ 12 vs. < 12 drinks/year), smoking status (current: smoked > 100 cigarettes and currently smoking; former: smoked > 100 cigarettes but quit; never: < 100 cigarettes), and physical activity. Medical history variables—determined through self‐reports, medication use, clinical assessments, and laboratory data—included hypertension, hyperlipidemia, CVD (e.g., heart failure, coronary artery disease, angina, myocardial infarction, and stroke), and cancer.

### 2.6. Statistical Analysis

Following NHANES analytic and reporting guidelines, we applied sample weights, stratification, and clustering in our analyses [36]. Participants were categorized into quartiles (Q1–Q4) based on the ln CALLY index. Descriptive statistics included interquartile ranges (IQRs) for continuous variables and frequencies with percentages for categorical variables. Differences among ln CALLY quartile groups were assessed using the Kruskal–Wallis test for continuous variables and the chi‐squared test with Rao and Scott’s second‐order correction for categorical variables, accounting for the complex survey design.

Multivariate Cox proportional hazards models were used to evaluate the associations between the CALLY index (both as a categorical and continuous variable) and ACM and CVM. Three models with increasing levels of adjustment were constructed. Model 1 assessed the univariate association between the CALLY index and outcomes. Model 2 adjusted for demographic variables, including age, gender, race/ethnicity, education level, marital status, and PIR. Model 3 further included behavioral factors, physical examination measures, and clinical comorbidities, including smoking status, alcohol use, BMI, physical activity, and history of hypertension, hyperlipidemia, CVD, and cancer. To address missing data and preserve sample size, multiple imputation by chained equations (MICE) was applied. Details regarding the proportion of missing covariates and the imputation methods are presented in Table S2. A linear trend test was conducted by including the median value of each ln CALLY index quartile as a continuous variable in the regression model. We calculated the generalized variance inflation factor (GVIF) for all variables in the analysis to evaluate multicollinearity. With all GVIF values below 2 (Table S1), no significant multicollinearity was detected. Subgroup analyses were conducted to verify the stability of the association between the CALLY index and ACM and CVM. Analyses were stratified by gender, race, education, marital status, PIR, smoking, alcohol use, BMI, physical activity, hypertension, hyperlipidemia, CVD history, and cancer history. Interaction terms between the CALLY index and various covariates were included, and interactions between subgroups were assessed using likelihood ratio tests. Estimates of survival probability were obtained through the Kaplan–Meier analysis. To explore potential nonlinear associations, restricted cubic spline (RCS) analysis was applied to examine the relationship between the CALLY index and the risks of ACM and CVM in individuals with diabetes or prediabetes.

To ensure the robustness of the results, multiple sensitivity analyses were conducted. First, individuals with incomplete covariate data were excluded to reduce the potential impact of missing information on the main outcomes. Second, considering the strong association between cancer and ACM in individuals with diabetes, participants who self‐reported a cancer diagnosis at baseline were removed to address possible confounding. Third, to mitigate reverse causality, those who died within the first 2 years of follow‐up were excluded using a delayed‐entry approach. Lastly, to minimize the effect of extreme values, participants with the ln CALLY index values beyond the mean ± 3 standard deviations were excluded. All statistical analyses were conducted using RStudio (version 4.5.0), with a two‐sided *p*‐value < 0.05 considered statistically significant.

## 3. Result

### 3.1. Characteristics of the Study Participants

The characteristics of these 8463 participants according to quartiles of the ln CALLY index are shown in Table 1. The median (IQR) age of the individuals was 53 (40,65) years, and 4486 (54%) were male. With the increase in the ln CALLY index, a greater proportion of individuals were male, above high school, and high income. Compared with individuals in the lower quartile, participants in the higher quartile were also more likely to be married or living with a partner, never smoke, and exhibit normal BMI and physical activity. Furthermore, individuals in the higher quartile showed a lower prevalence of hyperlipidemia, hypertension, and a history of cancer or CVD, as well as lower all‐cause or CVD mortality. These findings suggest a potential link between healthy lifestyle factors and increased CALLY index.

**Table 1:** Baseline characteristics according to ln CALLY index quartiles.

Characteristic	Overall *N* = 8463 (%)	Q1 N = 2229 (%)	Q2 N = 2164 (%)	Q3 N = 2072 (%)	Q4 N = 1998 (%)	*p* value
Age (years)	53 (40, 65)	53 (41, 65)	55 (42, 66)	54 (41, 66)	49 (34, 62)	< 0.001
Sex						< 0.001
Female	3977 (46)	1298 (59)	1107 (52)	859 (40)	713 (34)	
Male	4486 (54)	931 (41)	1057 (48)	1213 (60)	1285 (66)	
Race						< 0.001
Mexican American	1622 (9.2)	391 (8.5)	443 (9.8)	433 (10)	355 (8.6)	
Non‐Hispanic Black	1691 (11)	557 (15)	434 (12)	335 (8.9)	365 (11)	
Non‐Hispanic White	3554 (66)	967 (66)	915 (67)	892 (68)	780 (64)	
Other Race	1596 (13)	314 (11)	372 (12)	412 (13)	498 (17)	
Education						0.013
Above high school	3827 (54)	964 (50)	979 (53)	911 (54)	973 (58)	
High school	2072 (27)	566 (29)	506 (27)	518 (26)	482 (25)	
Under high school	2564 (19)	699 (21)	679 (20)	643 (19)	543 (17)	
Family PIR						< 0.001
High income	2369 (40)	512 (34)	571 (39)	615 (42)	671 (48)	
Low income	2688 (21)	797 (25)	709 (22)	607 (20)	575 (18)	
Medium income	3406 (38)	920 (41)	884 (40)	850 (38)	752 (34)	
Marital status						0.041
Together	5237 (66)	1299 (63)	1327 (65)	1334 (68)	1277 (66)	
Alone	3226 (34)	930 (37)	837 (35)	738 (32)	721 (34)	
Smoking						0.002
Former	2439 (29)	711 (32)	628 (30)	584 (29)	516 (26)	
Never	4424 (51)	1071 (48)	1108 (49)	1113 (53)	1132 (56)	
Now	1600 (19)	447 (20)	428 (21)	375 (18)	350 (18)	
Alcohol use						< 0.001
Former	1941 (19)	588 (22)	514 (22)	473 (18)	366 (16)	
Heavy	1483 (18)	339 (16)	379 (18)	351 (18)	414 (20)	
Mild	2802 (37)	659 (33)	706 (35)	705 (40)	732 (40)	
Moderate	1029 (14)	284 (15)	260 (13)	252 (13)	233 (13)	
Never	1208 (12)	359 (14)	305 (12)	291 (11)	253 (10)	
BMI						< 0.001
Normal/Underweight	1799 (21)	247 (10)	298 (12)	440 (20)	814 (41)	
Obese	3808 (46)	1458 (67)	1148 (56)	788 (38)	414 (22)	
Overweight	2856 (33)	524 (22)	718 (32)	844 (42)	770 (37)	
Recreational physical activity						< 0.001
Active	3179 (43)	651 (33)	757 (39)	821 (46)	950 (53)	
Inactive	5284 (57)	1578 (67)	1407 (61)	1251 (54)	1048 (47)	
Hypertension	4450 (48)	1344 (56)	1188 (51)	1068 (48)	850 (38)	< 0.001
Hyperlipidemia	6726 (79)	1824 (82)	1825 (85)	1661 (80)	1416 (71)	< 0.001
CVD	1304 (13)	445 (16)	329 (14)	303 (12)	227 (9.3)	< 0.001
Cancer	980 (12)	309 (14)	242 (11)	235 (12)	194 (10)	0.026
Follow‐up time (months)	109 (37, 147)	111 (38, 148)	89 (36, 147)	106 (34, 147)	113 (39, 145)	0.5
All‐cause mortality	1391 (12)	501 (17)	358 (13)	304 (11)	228 (8.9)	< 0.001
CVD mortality	470 (4.2)	167 (5.7)	118 (4.1)	108 (4.0)	77 (2.9)	< 0.001

*Note:* Q: quartile; ln: natural‐logarithm; CALLY: C‐reactive protein–albumin–lymphocyte index.

Abbreviations: BMI: body mass index; CVD: cardiovascular disease; PIR: poverty income ratio.

### 3.2. Associations Between the CALLY Index and Mortality

Over an average follow‐up period of 7.83 years, there were 1391 deaths, including 470 from cardiovascular causes. Table 2 presents the link between the CALLY index and mortality outcomes. After adjusting all interfering factors in model 3, it was negatively associated with ACM (HR = 0.83, 95% CI = 0.79–0.88) and CVM (HR = 0.82, 95% CI = 0.74–0.92). After adjusting for multiple variables in model 3, the hazard ratios (HRs) and 95% confidence intervals (CIs) for ACM across the lowest to highest quartiles of the ln CALLY index were 1.00 (reference), 0.68 (0.56–0.82), 0.62 (0.52–0.75), and 0.67 (0.55–0.81), with a significant linear trend (*p* < 0.001). For CVM, the HRs and 95% CIs were 1.00 (reference), 0.65 (0.46–0.92), 0.74 (0.53–1.02), and 0.66 (0.45–0.98), with no significant trend observed (*p* = 0.070). Weighted Kaplan–Meier curves showed higher ACM and CVM in individuals with a lower ln CALLY index (*p* < 0.001) (Figure 2). Weighted RCS analysis revealed a significant nonlinear, anti‐hook‐shaped association between ln CALLY index and ACM/CVM. Mortality decreased with increasing ln CALLY index and then rose slightly beyond a threshold (Figure 3).

**Table 2:** Hazard ratios and 95% confidence intervals for mortality according to ln CALLY index quartiles.

Variable	Model 1		Model 2		Model 3	
	HR (95% CI)	*p* value	HR (95% CI)	*p* value	HR (95% CI)	*p* value
*All-cause mortality*						
Ln CALLY	0.78 (0.74–0.83)	**< 0.001**	0.80 (0.76–0.85)	**< 0.001**	0.83 (0.79–0.88)	**< 0.001**

*Category*						
Quartile 1	—		—		—	
Quartile 2	0.75 (0.62–0.90)	**0.002**	0.65 (0.55–0.78)	**< 0.001**	0.68 (0.56–0.82)	**< 0.001**
Quartile 3	0.64 (0.53–0.76)	**< 0.001**	0.60 (0.51–0.72)	**< 0.001**	0.62 (0.52–0.75)	**< 0.001**
Quartile 4	0.52 (0.43–0.62)	**< 0.001**	0.62 (0.51–0.75)	**< 0.001**	0.67 (0.55–0.81)	**< 0.001**
P for trend		**< 0.001**		**< 0.001**		**< 0.001**

*Cardiovascular mortality*						
Ln CALLY	0.77 (0.70–0.84)	**< 0.001**	0.79 (0.71–0.88)	**< 0.001**	0.82 (0.74–0.92)	**< 0.001**

*Category*						
Quartile 1	—		—		—	
Quartile 2	0.75 (0.55–1.02)	0.065	0.66 (0.47–0.92)	**0.013**	0.65 (0.46–0.92)	**0.014**
Quartile 3	0.72 (0.55–0.94)	**0.016**	0.69 (0.50–0.94)	**0.018**	0.74 (0.53–1.02)	0.067
Quartile 4	0.51 (0.36–0.71)	**< 0.001**	0.61 (0.43–0.87)	**0.006**	0.66 (0.45–0.98)	**0.038**
P for trend		**< 0.001**		**0.01**		**0.07**

*Note:* Model 1 was unadjusted. Model 2 was adjusted for gender, age, race, education, marital status, and poverty income ratio. Model 3 was built upon Model 2, with additional adjustments for smoking, alcohol consumption, BMI, physical activity, hypertension, hyperlipidemia, history of CVD, and history of cancer. ln: natural‐logarithm; CALLY: C‐reactive protein–albumin–lymphocyte index. Bold values denote statistical significance (*p* < 0.05).

Abbreviations: BMI: body mass index; CI: confidence interval; CVD: cardiovascular disease; HR: hazard ratio.

Figure 2Kaplan–Meier curves showing survival probabilities among individuals with diabetes or prediabetes, stratified by quartiles of the ln CALLY index, with numbers at risk displayed below the curves. (a) All‐cause mortality. (b) Cardiovascular mortality. Abbreviations ln: natural logarithm; CALLY: C‐reactive protein–albumin–lymphocyte index; Q: quartile.

**Figure:**
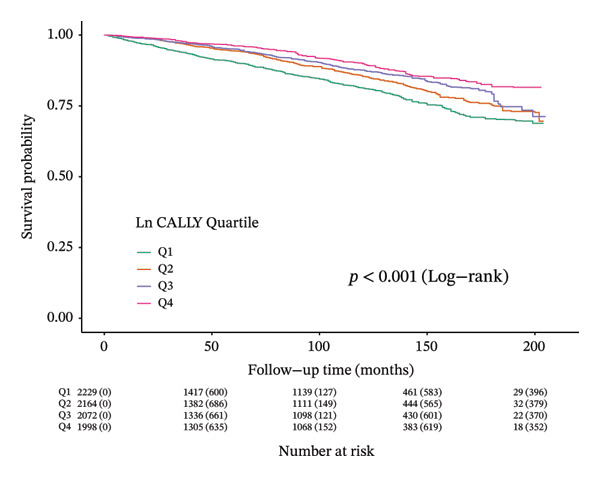
(a)

**Figure:**
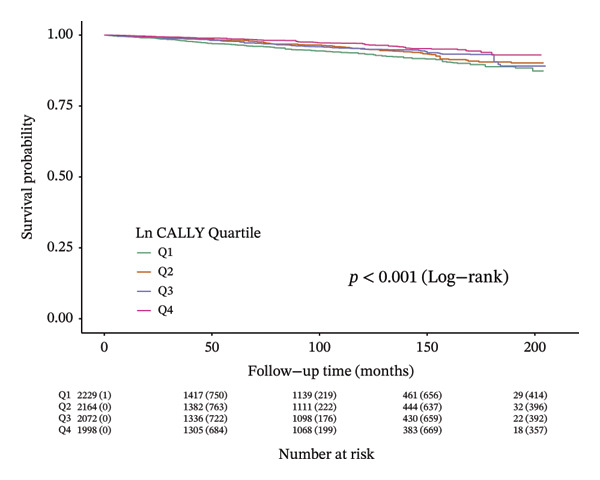
(b)

Figure 3Restricted cubic splines were utilized to evaluate the hypothesis of potential nonlinear relationships between the ln CALLY index and all‐cause (a) and cardiovascular (b) mortality in participants with diabetes or prediabetes. Abbreviations ln: natural logarithm; CALLY: C‐reactive protein–albumin–lymphocyte index; CI: confidence interval; HR: hazard model; BMI: body mass index; CVD: cardiovascular disease.

**Figure:**
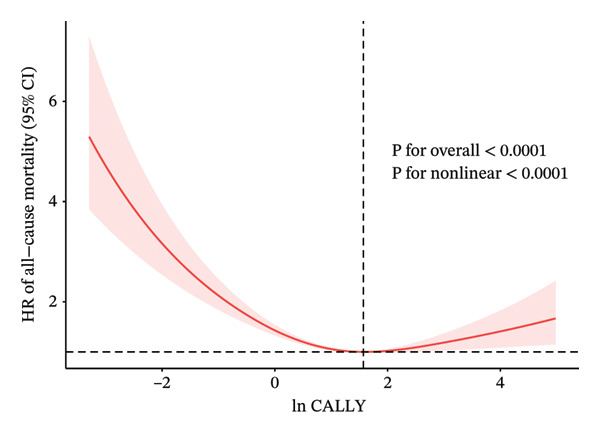
(a)

**Figure:**
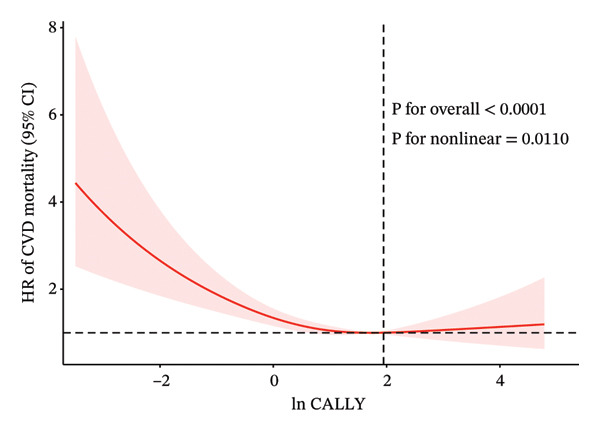
(b)

### 3.3. Subgroup Analysis

**Figure 4:**
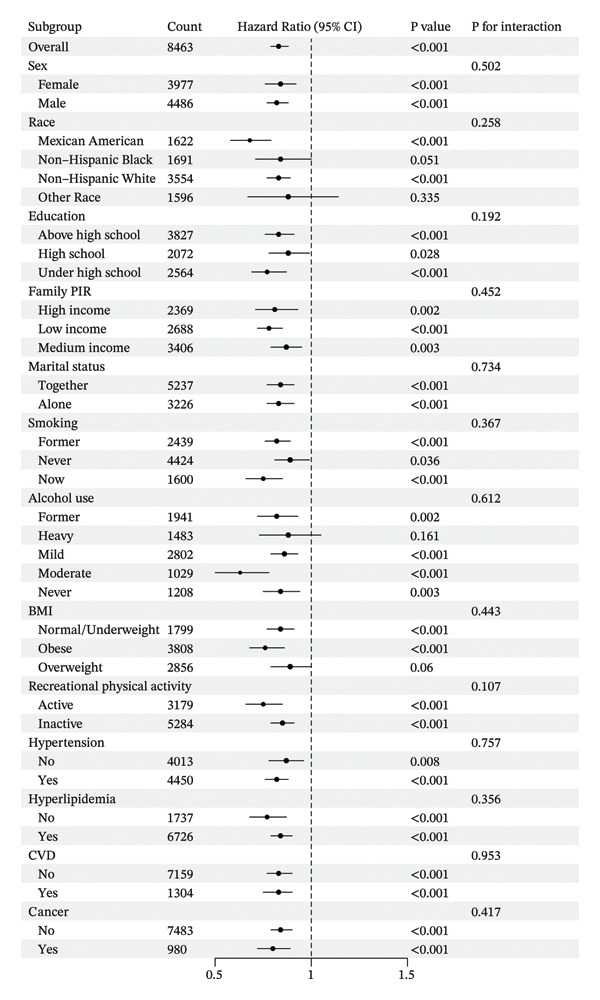
Subgroup analysis of the associations between the ln CALLY index and all‐cause mortality. Abbreviations ln: natural logarithm; CALLY: C‐reactive protein–albumin–lymphocyte index; BMI: body mass index; PIR: poverty income ratio; CVD: cardiovascular disease; CI: confidence interval.

**Figure 5:**
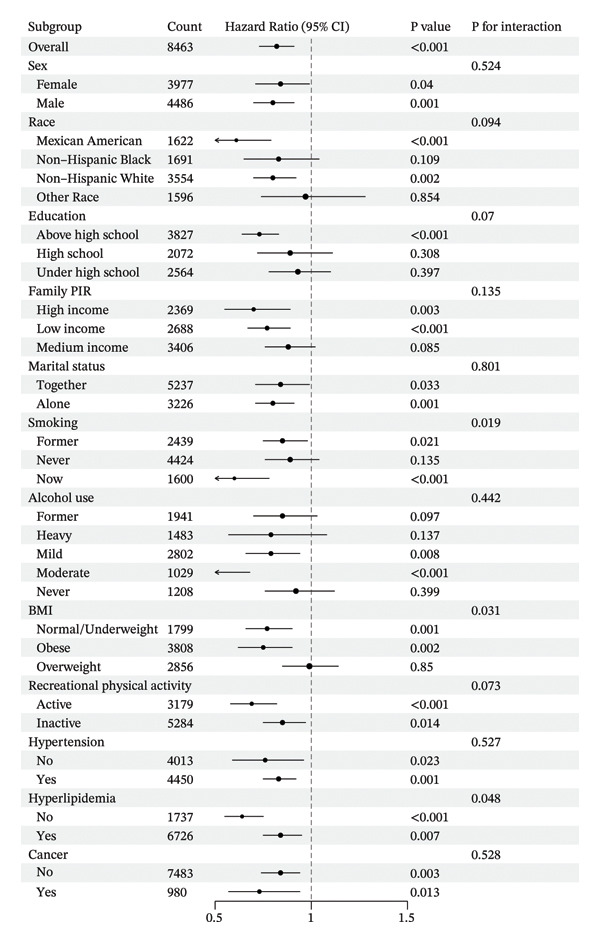
Subgroup analysis of the associations between the ln CALLY index and cardiovascular mortality. Abbreviations ln: natural logarithm; CALLY: C‐reactive protein–albumin–lymphocyte index; BMI: body mass index; PIR: poverty income ratio; CI: confidence interval.

Figures 4 and 5 show the stratification by gender, race, education, marital status, PIR, smoking status, alcohol consumption, physical activity, BMI, hypertension, hyperlipidemia, and history of CVD or cancer. Associations between the CALLY index and the risk of ACM in individuals with diabetes or prediabetes were consistent across the vast majority of subgroups, with a similar pattern observed for CVM. In subgroup analyses of CVD mortality, both smoking and BMI status showed significant interactions (P for interaction = 0.019 and 0.031, respectively). The association between the CALLY index and CVD mortality was more pronounced among current smokers and individuals with normal/underweight or obesity, suggesting that smoking and BMI may modify this relationship.

### 3.4. Sensitivity Analysis

In the sensitivity analysis, several exclusions were applied to assess the robustness of the findings. Excluding participants with missing baseline covariates reduced the sample to 6,934, with the association between the CALLY index and ACM and CVM remaining consistent (Table S3). Removing individuals with cancer resulted in a study population of 7,483, where adjusted analyses confirmed similar findings as the primary analysis (Table S4). After excluding participants from the initial 2 years of follow‐up, 7484 participants remained, with associations similar to those previously observed (Table S5). Finally, after removing extreme ln CALLY index values (mean ± 3 SD), 8417 participants were analyzed, with the associations yielding similar associations after adjusting for potential confounders (Table S6).

## 4. Discussion

To our knowledge, this study provides among the earliest prospective cohort evidence examining the association between the CALLY index and mortality outcomes in individuals with diabetes or prediabetes. Unlike previous isolated approaches using single biomarkers, the CALLY index better reflects the multifactorial influences on health outcomes in real‐world settings. Our findings revealed a significant inverse relationship between the CALLY index and ACM and CVM. In summary, these findings suggest that the CALLY index may serve as a valuable predictor of mortality in diabetes and prediabetes. The application of this composite index makes the research conclusions more consistent and reliable, which will have potential clinical utility in the future.

Diabetes is a chronic metabolic disease characterized by hyperglycemia and insulin resistance. Chronic hyperglycemia can lead to multiple organ injury. A common serious complication of diabetes is diabetic angiopathy. Among these, CVD—notably coronary artery disease and cerebrovascular disease—is the most common vascular complication encountered in clinical practice [37], whose pathophysiology primarily involves atherosclerosis. The CALLY index, composed of CRP, albumin, and lymphocyte count, serves as a comprehensive marker for assessing an individual’s immune, nutritional, and inflammatory status, while inflammation, nutrition, and immunity play important roles in the progression of CVD.

Chronic low‐grade inflammation represents a central pathophysiological mechanism in diabetes and a major contributor to diabetic vascular complications. Hyperglycemia activates inflammatory signaling pathways and cytokines, thereby sustaining a proinflammatory state [38–41]. This forms a vicious cycle of insulin resistance, β‐cell dysfunction, and atherosclerosis, driving the malignant progression of diabetes [42].

Nutritional status plays an important role in CVD development. Malnutrition may induce inflammatory responses, thereby increasing the risk of atherosclerosis [8, 43]. In patients with diabetes, multiple complications and chronic inflammation jointly predispose individuals to malnutrition through several biological mechanisms [44, 45]. These interconnected mechanisms collectively drive malnutrition‐inflammation‐atherosclerosis crosstalk in diabetes.

The immune system plays a central role in diabetes progression and contributes to atherosclerosis through bidirectional immune mechanisms under diabetic conditions. Hyperglycemia directly impairs innate (including macrophages, neutrophils, etc.) and adaptive(B and T cells) immune system, and further aggravates vascular inflammation [20]. This immunologic imbalance may exacerbate vascular inflammation and serve as a key mechanistic link between diabetes and atherosclerosis. On one hand, proinflammatory immune cells promote vascular inflammation and plaque formation through the secretion of immunoglobulins and proinflammatory cytokines [46, 47]. On the other hand, regulatory T and B cells exert protective effects by producing interleukin‐10, transforming growth factor‐β, and anti‐inflammatory immunoglobulin M [46, 48, 49].

Collectively, inflammation, malnutrition, and immune imbalance form a self‐reinforcing pathological network that accelerates the progression of diabetes and CVD, and the CALLY index—integrating CRP, serum albumin, and lymphocyte count—captures this complex internal milieu more comprehensively, offering potential advantages in prognostic accuracy and predictive value.

In our study, higher CALLY index values were generally associated with lower risks of ACM and CVM among patients with diabetes or prediabetes. This inverse association was observed in both continuous and categorical analyses, although statistical significance in the categorical models was limited to the second and fourth quartiles. Most previous studies on ACM and CVM in diabetic populations have focused on individual biomarkers, while comprehensive assessments incorporating immune, inflammatory, and nutritional status remain limited. Our findings underscore the prognostic superiority of the CALLY index, which integrates inflammatory (CRP), nutritional (albumin), and immunological (lymphocyte) pathways. CRP is a well‐established inflammatory marker and has been linked to atherosclerosis progression and increased CVD risk in patients with diabetes [13, 50]. Serum albumin reflects both nutritional and inflammatory status and has been independently associated with cardiovascular events and ACM [19, 51–54]. Lymphocytes play a key role in immune regulation, and lower lymphocyte counts have been associated with increased risks of ACM and cause‐specific mortality [55]. Consistently, higher values of related composite indices, such as the Prognostic Nutritional Index, have been associated with lower ACM in patients with type 2 diabetes [56]. Taken together, a higher CALLY index reflects more favorable inflammatory–nutritional–immune status, characterized by lower CRP levels, higher serum albumin levels, and higher lymphocyte counts, which is consistent with the observed reductions in ACM and CVM.

Previous studies have primarily investigated the prognostic value of the CALLY index in cancer patients, with higher levels generally indicating better outcomes [57–59]. Emerging evidence also suggests that the CALLY index is associated with various metabolic abnormalities, including hyperglycemia, hypertension, and hyperlipidemia [60]. More recently, the index has been applied to cardiovascular and cerebrovascular diseases, including myocardial infarction and stroke [61, 62], and several studies have reported that higher CALLY index levels are associated with lower risks of ACM and CVM in various populations [27, 29, 30]. These findings are largely in line with the results of our study, which demonstrated a similar inverse relationship between the CALLY index and both ACM and CVM in individuals with diabetes or prediabetes.

While previous results indicate an overall inverse association between the CALLY index and mortality risk, we further observed a nonlinear “reverse‐hook” curve, initially decreasing with rising CALLY index values and slightly increasing at extremely high levels. In diabetic individuals, this pattern may be attributed to hyperglycemia‐induced albumin glycation, leading to advanced glycation end‐products that impair immune regulation and promote inflammation [63] or the dual role of immune cells in the development of atherosclerosis [46, 48].

This study has several strengths. It is based on a large, nationally representative cohort with long‐term follow‐up, supporting the robustness and generalizability of the findings. In addition, multiple analytical approaches, including RCS modeling, subgroup analyses, and sensitivity analyses, were applied to enhance the reliability of the results. Several limitations should also be considered. The study population was derived from a U.S. cohort, which may limit generalizability to other populations. The CALLY index was assessed only at baseline, precluding evaluation of its longitudinal changes. Finally, as an observational study, causal relationships cannot be inferred, and residual confounding cannot be entirely excluded despite multivariable adjustment.

## 5. Conclusion

The CALLY index was significantly and negatively associated with both ACM and CVM among individuals with diabetes or prediabetes. As a simple and readily obtainable composite marker, the CALLY index holds potential as a useful tool for risk stratification and individualized intervention in this high‐risk population.

**Nomenclature**
ACM: all‐cause mortality
CVM: cardiovascular mortality
RCS: restricted cubic spline
CRP: C‐reactive protein
CVD: cardiovascular disease
ln: natural‐logarithm
CALLY: C‐reactive protein–albumin–lymphocyte index
BMI: body mass index
PIR: poverty income ratio
HR: hazard ratio
CI: confidence interval

## Author Contributions

Jie Li and Jing Yang conceptualized the research aims. Qian Liu, Renyang Liu, and Jie Li participated in data analysis and interpretation. Qian Liu and Renyang Liu wrote the first draft of the paper, and the other authors provided comments and approved the final manuscript. Qian Liu and Renyang Liu contributed equally to this study and share the first authorship. Jie Li and Jing Yang are corresponding authors.

## Funding

This research did not receive any specific grant from funding agencies in the public, commercial, or not‐for‐profit sectors.

## Ethics Statement

This study was performed in line with the principles of the Declaration of Helsinki. Approval was granted by the Ethics Committee of the National Center for Health Statistics. The patients/participants provided written informed consent to participate in this study.

## Conflicts of Interest

The authors declare no conflicts of interest.

## Supporting Information

Table S1: Assessment of multicollinearity among independent variables.

Table S2: The proportion of missing covariates and the imputation methods.

Table S3: Hazard ratios and 95% confidence intervals for mortality according to ln CALLY index quartiles after excluding participants with any missing covariate values at baseline.

Table S4: Hazard ratios and 95% confidence intervals for mortality according to ln CALLY index quartiles after excluding participants with self‐reported cancer at baseline.

Table S5: Hazard ratios and 95% confidence intervals for mortality according to ln CALLY index quartiles after excluding participants from the first 2 years of follow‐up.

Table S6: Hazard ratios and 95% confidence intervals for mortality according to ln CALLY index quartiles after excluding extreme values (mean ± 3 standard deviations) of the ln CALLY index.

## Supporting information


**Supporting Information** Additional supporting information can be found online in the Supporting Information section.

## Data Availability

Publicly available datasets were analyzed in this study. These data can be found at https://www.cdc. gov/nchs/nhanes/.
