# Supplementary material for: C‐Reactive Protein–Albumin–Lymphocyte Index as a Predictor of All‐Cause and Cardiovascular Mortality in Individuals With Diabetes or Prediabetes: A National Population‐Based Cohort Study
Source: Int J Endocrinol. 2026 Feb 9;2026:2774096. doi: 10.1155/ije/2774096 (PMC12884354; doi:10.1155/ije/2774096)
Supplement: Supplementary file 1 — Supporting Information Additional supporting information can be found online in the Supporting Information section. [file IJE-2026-2774096-s001.zip › Additional file1.docx]

**C-Reactive Protein-Albumin-Lymphocyte Index as a Predictor of All-Cause and Cardiovascular Mortality in Individuals with Diabetes or Prediabetes: A National Population-Based Cohort Study**

**Supplementary Information**

**Table S1.** Assessment of multicollinearity among independent variables.

**Table S2.** The proportion of missing covariates and the imputation methods.

**Table S3.** Hazard ratios and 95% confidence intervals for mortality according to ln CALLY index quartiles after excluding participants with any missing covariate values at baseline.

**Table S4.** Hazard ratios and 95% confidence intervals for mortality according to ln CALLY index quartiles after excluding participants with self-reported cancer at baseline.

**Table S5.** Hazard ratios and 95% confidence intervals for mortality according to ln CALLY index quartiles after excluding participants from the first 2 years of follow-up.

**Table S6.** Hazard ratios and 95% confidence intervals for mortality according to ln CALLY index quartiles after excluding extreme values (mean ± 3 standard deviations) of ln CALLY index.

**Additional file1: Table S1.** Assessment of multicollinearity among independent variables

| **Variables** | **GVIF** | **Df** | **GVIF1/2Df** |
| --- | --- | --- | --- |
| Ln CALLY | 1.7396 | 1 | 1.318956 |
| Gender | 2.211876 | 1 | 1.487225 |
| Age | 3.343958 | 1 | 1.828137 |
| Race | 4.027213 | 3 | 1.327964 |
| Education | 3.070762 | 2 | 1.515181 |
| Marital status | 1.982798 | 1 | 1.408123 |
| PIR | 5.88652 | 2 | 1.868285 |
| Smoking | 2.354347 | 2 | 1.339317 |
| Alcohol use | 3.090975 | 3 | 1.326331 |
| BMI | 2.497679 | 2 | 1.322059 |
| Physical activity | 1.701888 | 1 | 1.304573 |
| Hypertension | 2.144011 | 1 | 1.464279 |
| Hyperlipidemia | 1.553527 | 1 | 1.24519 |
| History of CVD | 2.013221 | 1 | 1.41893 |
| History of cancer | 1.776723 | 1 | 1.333951 |

Abbreviations: ln: natural-logarithm; CALLY: C-reactive protein-albumin-lymphocyte index; PIR: poverty income ratio; BMI: body mass index; CVD: cardiovascular diseases.

**Additional file1: Table S2.** The proportion of missing covariates and the imputation methods

| **Variable** | **Proportion of missing (%)** | **Imputation Methods** |
| --- | --- | --- |
| PIR | 9.10 | pmm |
| Alcohol use | 7.94 | pmm |
| BMI | 1.50 | pmm |
| Marital status | 1.78 | pmm |
| Smoking | 1.81 | pmm |
| Education | 0.15 | pmm |
| Hypertension | 0.01 | pmm |
| History of CVD | 2.66 | pmm |
| History of cancer | 2.78 | pmm |

Abbreviations: PIR: poverty income ratio; BMI: body mass index; CVD: cardiovascular diseases; pmm: predictive mean matching.

**Additional file1: Table S3.** Hazard ratios and 95% confidence intervals for mortality according to ln CALLY index quartiles after excluding participants with any missing covariate values at baseline

|  | **Model 1** | | **Model 2** | | **Model 3** | |
| --- | --- | --- | --- | --- | --- | --- |
| **Variable** | **HR (95% CI** | **P Value** | **HR (95% CI)** | **P Value** | **HR (95% CI)** | **P Value** |
| **All-cause mortality**  Ln CALLY | 0.80(0.75–0.86) | **<0.001** | 0.82 (0.77–0.87) | **<0.001** | 0.84(0.79–0.89) | **<0.001** |
| Category |  |  |  |  |  |  |
| Quartile 1 | — |  | — |  | — |  |
| Quartile 2  Quartile 3 | 0.78 (0.64–0.95)  0.69 (0.57–0.84) | **0.014**  **<0.001** | 0.67 (0.55–0.82)  0.64 (0.53–0.76) | **<0.001**  **<0.001** | 0.67 (0.54–0.83)  0.65 (0.54–0.78) | **<0.001**  **<0.001** |
| Quartile 4 | 0.55 (0.45–0.67) | **<0.001** | 0.65 (0.53–0.80) | **<0.001** | 0.68 (0.55–0.85) | **<0.001** |
| P for trend |  | **<0.001** |  | **<0.001** |  | **0.001** |
| **Cardiovascular mortality**  Ln CALLY | 0.77(0.69–0.85) | **<0.001** | 0.78 (0.68–0.88) | **<0.001** | 0.80 (0.71–0.90) | **<0.001** |
| Category |  |  |  |  |  |  |
| Quartile 1 | — |  | — |  | — |  |
| Quartile 2  Quartile 3 | 0.75 (0.55–1.04)  0.75 (0.55–1.02) | **0.085**  **0.069** | 0.64 (0.46–0.90)  0.68(0.48–0.97) | **0.010**  **0.035** | 0.60(0.43–0.85)  0.71(0.49–1.02) | **0.004**  0.068 |
| Quartile 4 | 0.49 (0.34–0.70) | **<0.001** | 0.58(0.38–0.86) | **0.007** | 0.61 (0.40–0.95) | **0.027** |
| P for trend |  | **<0.001** |  | **0.014** |  | 0.066 |
| Model 1 was unadjusted.  Model 2 was adjusted for gender, age, race, education, marital status, and poverty income ratio.  Model 3 was built upon Model 2, with additional adjustments for smoking, alcohol consumption, BMI, physical activity, hypertension, hyperlipidemia, history of CVD, and history of cancer.  Abbreviations: ln: natural-logarithm; CALLY: C-reactive protein-albumin-lymphocyte index; HR: hazard ratio; CI: confidence interval; BMI: body mass index; CVD: cardiovascular disease.  **Additional file1: Table S4.**  Hazard ratios and 95% confidence intervals for mortality according to ln CALLY index quartiles after excluding participants with self-reported cancer at baseline   \|  \| **Model 1** \| \| **Model 2** \| \| **Model 3** \| \| \| --- \| --- \| --- \| --- \| --- \| --- \| --- \| \| **Variable** \| **HR (95% CI)** \| **P Value** \| **HR (95% CI)** \| **P Value** \| **HR (95% CI)** \| **P Value** \| \| **All-cause mortality**  Ln CALLY \| 0.79(0.75–0.85) \| **<0.001** \| 0.81 (0.76–0.87) \| **<0.001** \| 0.84 (0.79–0.90) \| **<0.001** \| \| Category \|  \|  \|  \|  \|  \|  \| \| Quartile 1 \| — \|  \| — \|  \| — \|  \| \| Quartile 2 \| 0.80(0.64–0.99) \| **0.039** \| 0.68 (0.55–0.85) \| **<0.001** \| 0.68(0.54–0.86) \| **0.001** \| \| Quartile 3  Quartile 4 \| 0.64 (0.53–0.78)  0.55 (0.45–0.67) \| **<0.001**  **<0.001** \| 0.60 (0.49–0.74)  0.65 (0.52–0.81) \| **<0.001**  **<0.001** \| 0.63 (0.51–0.78)  0.70 (0.56–0.88) \| **<0.001**  **0.002** \| \| P for trend \|  \| **<0.001** \|  \| **<0.001** \|  \| **0.005** \| \| **Cardiovascular mortality**  Ln CALLY \| 0.78 (0.70–0.87) \| **<0.001** \| 0.80 (0.72–0.90) \| **0<0.001** \| 0.84 (0.75–0.95) \| **0.005** \| \| Category \|  \|  \|  \|  \|  \|  \| \| Quartile 1 \| — \|  \| — \|  \| — \|  \| \| Quartile 2 \| 0.81(0.58–1.15) \| 0.237 \| 0.70 (0.47–1.02) \| 0.063 \| 0.68 (0.47–1.00) \| **0.047** \| \| Quartile 3  Quartile 4 \| 0.71(0.52–0.97)  0.51 (0.35–0.75) \| **0.030**  **<0.001** \| 0.68 (0.47–0.97)  0.60(0.40–0.91) \| **0.032**  **0.017** \| 0.75 (0.52–1.10)  0.68 (0.44–1.05) \| 0.144  0.085 \| \| P for trend \|  \| **<0.001** \|  \| **0.016** \|  \| 0.123 \| \| Model 1 was unadjusted.  Model 2 was adjusted for gender, age, race, education, marital status, and poverty income ratio.  Model 3 was built upon Model 2, with additional adjustments for smoking, alcohol consumption, BMI, physical activity, hypertension, hyperlipidemia, history of CVD, and history of cancer.  Abbreviations: ln: natural-logarithm; CALLY: C-reactive protein-albumin-lymphocyte index; HR: hazard ratio; CI: confidence interval; BMI: body mass index; CVD: cardiovascular disease. \| \| \| \| \| \| \| | | | | | | |

**Additional file 1: Table S5** Hazard ratios and 95% confidence intervals for mortality according to ln CALLY index quartiles after excluding participants from the first 2 years of follow-up

|  | **Model 1** | | **Model 2** | | **Model 3** | |
| --- | --- | --- | --- | --- | --- | --- |
| **Variable** | **HR (95% CI)** | **P Value** | **HR (95% CI)** | **P Value** | **HR (95% CI)** | **P Value** |
| **All-cause mortality**  **Ln CALLY** | 0.82(0.78–0.87) | **<0.001** | 0.85 (0.80–0.90) | **<0.001** | 0.88 (0.83–0.93) | **<0.001** |
| Category |  |  |  |  |  |  |
| Quartile 1 | — |  | — |  | — |  |
| Quartile 2 | 0.86 (0.71–1.03) | 0.099 | 0.73(0.61–0.88) | **<0.001** | 0.75(0.61–0.91) | **0.004** |
| Quartile 3  Quartile 4 | 0.69(0.57–0.83)  0.58 (0.48–0.70) | **<0.001**  **<0.001** | 0.65 (0.54–0.78)  0.69(0.57–0.84) | **<0.001**  **<0.001** | 0.67 (0.55–0.81)  0.74(0.61–0.90) | **<0.001**  **0.003** |
| P for trend |  | **<0.001** |  | **<0.001** |  | **0.003** |
| **Cardiovascular mortality**  Ln CALLY | 0.78 (0.71–0.86) | **<0.001** | 0.80 (0.71–0.90) | **<0.001** | 0.83 (0.73–0.93) | **0.002** |
| Category |  |  |  |  |  |  |
| Quartile 1 | — |  | — |  | — |  |
| Quartile 2 | 0.82(0.58–1.16) | 0.269 | 0.71 (0.49–1.03) | 0.069 | 0.70 (0.47–1.03) | 0.071 |
| Quartile 3  Quartile 4 | 0.69(0.52–0.92)  0.50 (0.32–0.72) | **0.011**  **<0.001** | 0.66 (0.47–0.93)  0.60 (0.40–0.89) | **0.018**  **0.011** | 0.71 (0.49–1.02)  0.65 (0.43–0.97) | 0.064  **0.034** |
| P for trend |  | **<0.001** |  | **0.010** |  | **0.040** |
| Model 1 was unadjusted.  Model 2 was adjusted for gender, age, race, education, marital status, and poverty income ratio.  Model 3 was built upon Model 2, with additional adjustments for smoking, alcohol consumption, BMI, physical activity, hypertension, hyperlipidemia,  history of CVD, and history of cancer.  Abbreviations: ln: natural-logarithm; CALLY: C-reactive protein-albumin-lymphocyte index; HR: hazard ratio; CI: confidence interval; BMI: body mass index; CVD: cardiovascular disease. | | | | | | |

**Additional file1: Table S6.** Hazard ratios and 95% confidence intervals for mortality according to ln CALLY index quartiles after excluding extreme values (mean ± 3 standard deviations) of ln CALLY index

|  | **Model 1** | | **Model 2** | | **Model 3** | |
| --- | --- | --- | --- | --- | --- | --- |
| **Variable** | **HR (95% CI)** | **P Value** | **HR (95% CI)** | **P Value** | **HR (95% CI)** | **P Value** |
| **All-cause mortality**  Ln CALLY | 0.79 (0.75–0.84) | **<0.001** | 0.82 (0.77–0.87) | **<0.001** | 0.84 (0.79–0.90) | **<0.001** |
| Category |  |  |  |  |  |  |
| Quartile 1 | — |  | — |  | — |  |
| Quartile 2 | 0.78 (0.65–0.95) | **0.012** | 0.70 (0.58–0.84) | **<0.001** | 0.71 (0.58–0.87) | **0.001** |
| Quartile 3  Quartile 4 | 0.66 (0.55–0.79)  0.53 (0.44–0.64) | **<0.001**  **<0.001** | 0.63 (0.53–0.75)  0.65 (0.54–0.79) | **<0.001**  **<0.001** | 0.65 (0.54–0.78)  0.70 (0.57–0.85) | **<0.001**  **<0.001** |
| P for trend |  | **<0.001** |  | **<0.001** |  | **<0.001** |
| **Cardiovascular mortality**  Ln CALLY | 0.79(0.72–0.86) | <0.001 | 0.81 (0.73–0.91) | **<0.001** | 0.84 (0.75–0.95) | **0.005** |
| Category |  |  |  |  |  |  |
| Quartile 1 | — |  | — |  | — |  |
| Quartile 2 | 0.79 (0.59–1.08) | 0.141 | 0.71 (0.52–0.98) | **0.038** | 0.71 (0.51–0.98) | **0.039** |
| Quartile 3  Quartile 4 | 0.74(0.56–0.97)  0.53 (0.38–0.74) | **0.029**  **<0.001** | 0.71 (0.52–0.97)  0.65 (0.46–0.92) | **0.031**  **0.014** | 0.77 (0.55–1.07)  0.70 (0.48–1.03) | 0.121  0.073 |
| P for trend |  | **<0.001** |  | **0.019** |  | 0.119 |
| Model 1 was unadjusted.  Model 2 was adjusted for gender, age, race, education, marital status, and poverty income ratio.  Model 3 was built upon Model 2, with additional adjustments for smoking, alcohol consumption, BMI, physical activity, hypertension, hyperlipidemia, history of CVD, and history of cancer.  Abbreviations: ln: natural-logarithm; CALLY: C-reactive protein-albumin-lymphocyte index; HR: hazard ratio; CI: confidence interval; BMI: body mass index; CVD: cardiovascular disease. | | | | | | |
